# Glucose but not insulin or insulin resistance is associated with memory performance in middle-aged non-diabetic women: a cross sectional study

**DOI:** 10.1186/s13098-015-0014-7

**Published:** 2015-03-15

**Authors:** Anna Backeström, Sture Eriksson, Lars-Göran Nilsson, Tommy Olsson, Olov Rolandsson

**Affiliations:** Department of Public Health and Clinical Medicine, Family Medicine, Umeå University, S-901 87 Umeå, Sweden; Department of Community Medicine and Rehabilitation, Geriatric Medicine, Umeå University, S-901 85 Umeå, Sweden; Department of Psychology, Stockholm University, S-106 91 Stockholm, Sweden; Department of Public Health and Clinical Medicine, Medicine, Umeå University, S-901 87 Umeå, Sweden

**Keywords:** Cognition, Memory, Insulin, Insulin resistance, HOMA-IR, Glucose, Episodic memory, Semantic memory

## Abstract

**Background:**

Elevated concentrations of plasma glucose appear to play a role in memory impairment, and it has been suggested that insulin might also have a negative effect on cognitive function. Our aim was to study whether glucose, insulin or insulin resistance are associated with episodic or semantic memory in a non-diabetic and non-demented population.

**Methods:**

We linked and matched two population-based data sets identifying 291 participants (127 men and 164 women, mean age of 50.7 ± 8.0 years). Episodic and semantic memory functions were tested, and fasting plasma insulin, fasting plasma glucose, and 2-hour glucose were analysed along with other potential influencing factors on memory function. Since men and women display different results on memory functions they were analysed separately. Insulin resistance was calculated using the HOMA-IR method.

**Results:**

A higher fasting plasma glucose concentration was associated with lower episodic memory in women (r = −0.08, 95% CI −0.14; −0.01), but not in men. Plasma insulin levels and insulin resistance were not associated with episodic or semantic memory in women or in men after adjustments for age, fasting glucose, 2-hour glucose, BMI, education, smoking, cardiovascular disease, hypertension, cholesterol, and physical activity.

**Conclusions:**

This indicates that fasting glucose but not insulin, might have impact on episodic memory in middle-aged women.

## Background

Type 2 diabetes is associated with increased risk for cognitive decline [1] and dementia [2], including Alzheimer’s disease [3]. The mechanisms behind these associations, however, are not fully understood. One reason for this association could be cardiovascular disease [4] and another cause might be the effects of metabolic perturbations [1].

High concentrations of glucose appear to play an important role in cognitive dysfunction [5] and dementia [6], and previous studies have revealed that cognitive impairments can occur even before the diagnosis of type 2 diabetes, i.e., in a pre-diabetic stage [7]. In line with this, we have previously reported that increased plasma glucose concentrations in non-diabetic women were associated with impairments in episodic memory [8]. It is well known that men and women display different results on different memory functions [9,10]. Thus it is of importance to also analyse men and women separately.

Before the onset of type 2 diabetes, the concentrations of insulin in the plasma increase as a result of insulin resistance. Some studies have indicated that elevated insulin concentrations [11-13] and insulin resistance [12,13] are associated with an increased risk of Alzheimer’s disease and impairments in cognitive function [11]. It is still unclear, however, whether insulin concentrations or insulin resistance play a role in memory function in the normoglycemic population [14,15].

Insulin influences the central nervous system as a neuromodulator [16] and regulates energy metabolism and neurotransmission [16,17]. Insulin crosses the blood brain barrier via an insulin receptor-mediated transport process [16,18] and insulin receptors are located in the medial temporal structures that support memory function [16,17]. Insulin might also affect memory through its inhibition of insulin degrading enzyme that in turn leads to a failure to degrade amyloid-β, a hallmark of Alzheimer’s disease [19].

To determine the possible associations between glucose, insulin, and development of impaired memory function, it is useful to study persons before the onset of diabetes or dementia to minimize the confounding effects of these diseases. Thus, our aim was to investigate whether insulin resistance, elevated concentrations of insulin or glucose were associated with impaired episodic and/or semantic memory in a non-diabetic and non-demented population.

## Methods

### Study population

We used data from two population-based surveys. The Betula Prospective Cohort Study (n = 3,527) investigated how memory functions and health develop over the adult life span [20]. The database with the participants in the Betula study was linked to the database of participants in the Västerbotten Intervention Program (VIP) (n = 72,861). The VIP is a health survey to which all inhabitants in the county of Västerbotten in northern Sweden have been invited to participate at the ages of 40, 50, and 60 years since 1987 [21].

In the Betula study years of formal education and any history of cardiovascular events were collected by questionnaire. Episodic- and semantic memory tests were performed. Episodic memory test included test of face/name recognition, sentence learning with encoding enactment, sentence learning without enactment, word recall, learning of facts, prospective memory, and memory for activities. Semantic memory included vocabulary, general knowledge, and word fluency tests. The procedures of the tests have been described in detail elsewhere [20,22].

In the VIP study weight and height were measured and body mass index (BMI; kg/m^2^) was calculated. Smokers were defined as those reporting daily smoking or “occasional smoking” and ex-smokers were classified as non-smokers. Blood pressure was measured with participants in a supine position after 5 minutes of rest. Hypertension was defined as self-reported hypertension, the use of anti-hypertensive medication, or systolic blood pressure ≥140 mmHg and/or diastolic blood pressure ≥90 mmHg.

Fasting plasma glucose and total fasting plasma cholesterol concentrations were measured in capillary plasma on a Reflotron bench-top analyser (Boeringer Mannheim GmbH, Mannheim, Germany) after an overnight fast. An oral glucose tolerance test was performed to measure 2-hour plasma glucose (2hPG) after a 75 g glucose load. Fasting plasma insulin was analysed (Roche Cobas e601 analyser, Roche Diagnostics, Diamond Diagnostics, Holliston, MA), and the homeostasis model assessment insulin-resistance (HOMA-IR) was used to calculate insulin resistance [23].

Physical activity was assessed from a questionnaire in the VIP that asked, “How often have you trained in an exercise outfit in the last three months in order to improve your fitness and/or to feel good?” The five response options were 1) never, 2) occasionally, not regularly, 3) once weekly, 4) two to three times per week, and 5) more than three times per week. The answers were categorized into a low activity group (responses 1 and 2) and a high activity group (responses 3–5).

**Figure 1:**
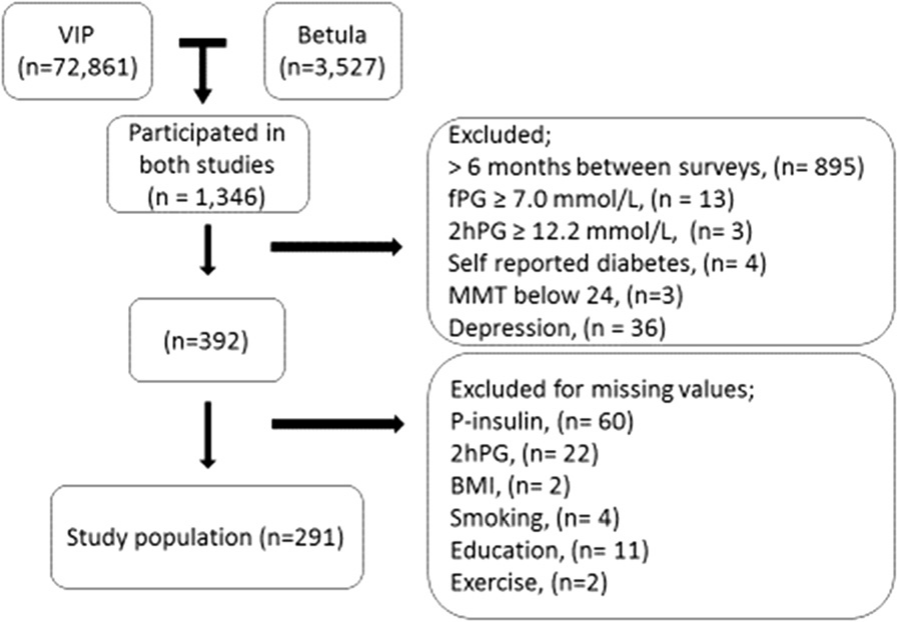
**Flow-chart of the study population.**

A flow-chart of the population is presented in Figure 1. We identified 1,346 individuals who had participated in both studies. To limit the risk of changes in metabolic variables due to the difference in time, we excluded participants with more than 6 months between surveys. Participants with fasting plasma glucose (fPG) ≥7.0 mmol/L or a glucose concentration two hours after glucose administration (2hPG) ≥12.2 mmol/L were considered to have diabetes and were excluded, as were those who reported that they had diabetes. We screened for dementia with the Mini Mental State Examination (MMSE) [24] and a score below 24 lead to exclusion. Participants filled in a screening test for depression (Centre for Epidemiologic Studies Depression Scale; CES-D) [25], and a score above 15 lead to exclusion, leaving 392 persons. Fasting plasma insulin concentrations (fPI) were available for 333 of these individuals. One insulin value was excluded as an error value (754 pmol/L). After correction for missing values in the variables for the potential influencing factors of memory it remained 291 participants in the study population (164 women and 127 men with a mean age of 50.7 ± 8.0 years). Forty-eight participants who lacked information on CES-D remained in the study population. To investigate if there was any signs of selection bias we compared years of education and weight between the first sample of 1346 persons (mean years of education 11.8 (SD 3.9) years; mean weight 74.3 (SD 13.3) kg) and our study sample (n = 291; mean years of education. 11.5 (SD 3.9) years; weight 74.0 (SD 12.8) kg; p > 0.05 for both comparisons).

The participants gave their informed consent, and we obtained approval from the ethics committee in Umeå, Sweden.

### Statistical analyses

In the statistical analyses descriptive data are presented as mean values with standard deviations (SD), numbers (n), and proportions (%). We standardized the memory testing data using the mean values and SD from the youngest age group to compute a composite z-score (z = (score - 40-year-old mean) / 40-year-old SD). Statistical significance of the means and proportions between the groups was tested for continuous and categorical variables by Student’s *t*-tests and chi-square tests, respectively. Pearson’s correlation analyses were performed. A difference was regarded as statistically significant when p <0.05.

We performed a partial least square regression analysis (PLS) [26] to examine the independent effects of glucose, insulin, and insulin resistance on episodic and semantic memory with adjustments for age, BMI, education level, smoking, cardiovascular disease, hypertension, cholesterol, and physical activity. PLS is a multivariate method that is used to best estimate the importance of the potential covariates in the model by determine how multiple x-variables co-vary with y-variables. Thus, PLS does not require independence among measures and is, therefore, able to deal with multicolinearity better than many other regression models [27]. Potential influencing factors were chosen because of their possible association to memory function based on previous findings. All variables were scaled to unit variance and centered before being introduced into the model. The jack-knife technique [28] was used to calculate the standard error and to estimate the 95% confidence intervals (CI) for the PLS- regression coefficients (r).

IBM SPSS Statistics 19.0 and the R: A language and environment for statistical computing [29] was used for statistical calculation.

## Results

Women had higher 2hPG than men, but there were no differences in fasting glucose or fasting insulin (Table 1). More men than women had hypertension. Women had better scores for both episodic and semantic memory compared to men, and these gender differences in memory function were explained by better results in the younger female age groups (Table 1). Due to the difference between men and women in the dependent variables, we stratified all of the data for gender.

**Table 1:** **Characteristics of the study population by gender**

	**Women**	**Men**	**p**	**All**
**n**	**164**	**127**	**NS**	**291**
Time between surveys (days)	13.9 (68.5)	17.6 (70.2)	NS	15.5 (69.1)
Age (years)	51.0 (8.1)	50.3 (8.0)	NS	50.7 (8.0)
40 years (n)	46	38	NS	84
50 years (n)	55	47	NS	102
60 years (n)	63	42	NS	105
fP-glucose (mmol/L)	5.2 (0.54)	5.3 (0.56)	NS	5.3 (0.55)
2hP-glucose (mmol/L)	6.8 (1.26)	6.4 (1.48)	0.007	6.6 (1.37)
fP-insulin (pmol/L)	54.9 (61.51)	47.6 (24.58)	NS	51.7 (49.00)
HOMA-IR	1.03 (1,11)	0.90 (0.47)	NS	0.97 (0.89)
Hypertension (%)	27	39	0.023	32
Cholesterol (mmol/L)	5.9 (1.29)	5.9 (1.18)	NS	5.9 (1.24)
BMI	25 (4.2)	26 (3.0)	NS	26 (3.7)
Educational level (years)	12 (3.8)	12 (3.9)	NS	12 (3.9)
Smoking (%)	20	26	NS	23
Cardiovascular disease (%)	27	20	NS	24
Exercise group (high) (%)	35	31	NS	33
Episodic memory (score)	7.29 (1.39)	6.62 (1.30)	<0.001	7.00 (1.39)
40 years	7.92 (1.41)	6.69 (1.20)	<0.001	7.36 (1.45)
50 years	7.39 (1.25)	6.75 (1.43)	0.019	7.09 (1.36)
60 years	6.75 (1.30)	6.42 (1.25)	NS	6.62 (1.29)
Semantic memory (score)	15.96 (2.82)	15.20 (3.03)	0.028	15.6 (2.94)
40 years	16.86 (2.10)	15.56 (2.07)	0.005	16.27 (2.17)
50 years	15.86 (3.07)	15.67 (2.90)	NS	15.77 (2.98)
60 years	15.39 (2.93)	14.33 (3.72)	NS	14.96 (3.30)

Data are shown as numbers (n), proportions (%), or mean values with the standard deviation in parentheses. P-values are given for differences between genders. NS means not significant. HOMA-IR is a calculated value for insulin resistance.

In the correlation analysis, episodic memory in women was correlated with exercise and educational level and inversely correlated with older age, fPG, 2hPG, fPI, HOMA-IR, previous incidence of cardiovascular disease (CVD), hypertension and cholesterol (Table 2). Years of education correlated with episodic memory performance in men. BMI and cholesterol values were inversely correlated (Table 2).

**Table 2:** **Correlation between episodic and semantic memory and potential influencing factors by gender**

	**Age**	**fPG**	**2hPG**	**fPI**	**HOMAIR**	**BMI**	**Education**	**Smoking**	**CVD**	**HT**	**Cholesterol**	**Exercise**
*Women*												
Episodic	−0.34‡	−0.28‡	−0.24†	−0.19*	−0.20*	−0.11	0.38‡	0.06	−0.18*	−0.22†	−0.24†	0.18*
Semantic	−0.21†	−0.09	−0.19*	−0.08	−0.08	−0.12	0.55‡	0.05	−0.22†	−0.21†	−0.19*	0.16*
*Men*												
Episodic	−0.08	0.08	−0.02	−0.14	−0.13	−0.22†	0.44‡	−0.06	−0.01	−0.12	−0.22*	0.17
Semantic	−0.16*	0.14	−0.001	−0.13	−0.12	−0.29‡	0.59‡	−0.08	−0.07	−0.01	−0.04	0.06

*p <0.05, † p <0.01, ‡ p <0.001.

Episodic and semantic memory, age, fasting plasma glucose (fPG), glucose after 2 hours glucose load (2hPG), fasting plasma insulin (fPI), insulin resistance (HOMA-IR), BMI, years in formal education, smoking, cardiovascular disease (CVD), hypertension (HT), cholesterol, and physical activity (exercise).

Semantic memory in women was correlated with exercise and inversely correlated with age, 2hPG, previous CVD, hypertension and cholesterol. (Table 2). In men semantic memory was inversely correlated with age and BMI. Education was correlated with semantic memory in both women and men (Table 2).

We used two matrix components in the PLS analysis explaining 29.3% in women and 24.7% in men of the variance for the dependent variable episodic memory, and 31.4% in women and 37.3% in men of the variance for the dependent variable semantic memory. We included age, fasting glucose, 2-hour glucose, fasting insulin, HOMA-IR, BMI, education, smoking, cardiovascular disease, hypertension, cholesterol, and physical activity as potential influencing factors of memory.

**Figure 2:**
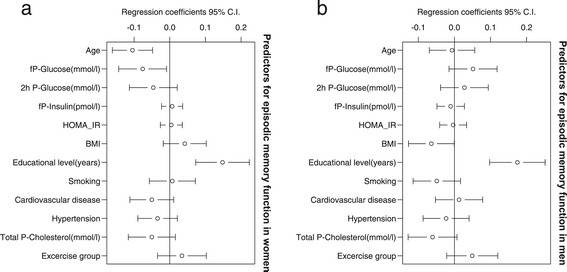
**Episodic memory in women and men in a PLS regression analysis. (a)** Episodic memory in women. The association between episodic memory and age, glucose, 2-hour glucose, insulin, insulin resistance (HOMA-IR), BMI, education, smoking, CVD, hypertension, cholesterol, and exercise group in women. **(b)** Episodic memory in men. The association between episodic memory and age, glucose, 2-hour glucose, insulin, insulin resistance (HOMA-IR), BMI, education, smoking, CVD, hypertension, cholesterol, and exercise group in men.

**Figure 3:**
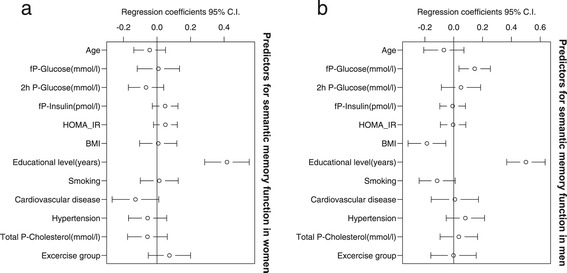
**Semantic memory in women and men in a PLS regression analysis. (a)** Semantic memory in women. The association between semantic memory and age, glucose, 2-hour glucose, insulin, insulin resistance (HOMA-IR), BMI, education, smoking, CVD, hypertension, cholesterol, and exercise group in women. **(b)** Semantic memory in men. The association between semantic memory and age, glucose, 2-hour glucose, insulin, insulin resistance (HOMA-IR), BMI, education, smoking, CVD, hypertension, cholesterol, and exercise group in men.

Poorer episodic memory function in women was associated with a higher glucose concentration (r = −0.08, 95% CI −0.14; −0.01) and a higher age (Figure 2a). Men with a higher BMI had lower scores on episodic memory (r = −0.06, 95% CI −0.13; −0.0005) (Figure 2b) and semantic memory (r = −0.19, 95% CI −0.32; −0.055) (Figure 3b.). Higher concentrations of glucose in men were associated with better performance on semantic memory tests (r = 0.14, 95% CI 0.04; 0.25) (Figure 3b). Years of education were a predictor for better episodic and semantic memory scores for both women (Figures 2a and 3a) and for men (Figures 2b and 3b).

Fasting insulin or HOMA-IR was not associated to episodic or semantic memory in women or in men in the PLS regression analysis.

## Discussion

Fasting plasma glucose was associated with poorer episodic memory in women. In contrast, we found no significant association between insulin concentrations or insulin resistance and episodic or semantic memory. This suggests that elevated glucose concentrations might influence memory function more, or earlier, than insulin.

A few studies have investigated the association between glucose, insulin, insulin resistance, and memory function in non-diabetic middle-aged individuals. When comparing different studies, it is important to take into account the effect of age on cognitive function because age is a possible confounder in the association between insulin, glucose, and memory [30,31]. We included 40, 50 and 60-year old volunteers in our study, i.e., the mean age was 50.7 (8.0) years.

The present results are in line with Messier et al. [32] who showed that higher blood glucose, but not insulin, was associated with poorer episodic memory in a population of 20-year-old students. Our findings are also corroborated by Sanz et al. [15] who found that neither increased fasting insulin nor insulin resistance were associated with episodic memory or semantic memory in a study population with a mean age of 50 years. Our study is in contrast to studies with older participants. For example, Bruehl et al. [33] showed that non-diabetic participants, with an average age of 63 years and with increased insulin resistance, showed poorer episodic memory. Tan et al. [14] found in the Framingham offspring study that high HOMA-IR and fasting insulin were related to reduced episodic memory among participants of about 61 years of age who did not have clinical diabetes. In a study by Kim et al. [34] a significant decrease in semantic memory was found in non-diabetic participants of about 71 years of age, and with mild cognitive impairments (MCI). However, this was only true for those who were APOE4 positive. Finally, Benedict et al. [35] found that insulin resistance was associated with poorer semantic memory performance in a population of 75 year olds. The discrepancy between our study and the four latter studies may be due to increased prevalence of metabolic and structural changes in older age [36].

Older ages in the latter studies are also associated to more components of the metabolic syndrome i.e. hyperglycemia, hyperinsulinemia, obesity, hypertension and hyperlipidemia. Our sample is relatively young, which may explain why we have only found an association to parts of the metabolic syndrome. It is possible that the increased insulin levels and/or insulin resistance could be more important for other cognitive profiles than those assessed in our study.

We found a difference in memory function between men and women. Women generally perform better than men on episodic memory verbal tasks and non-verbal tasks, e.g., face recognition [9,10], but there is currently no established explanation for this gender effect. The gender differences in episodic memory and semantic memory in our study were only seen in younger participants, i.e., premenopausal women, which could support the theory of an estrogen effect. The effects of sex hormones are commonly regarded as mediators of the gender difference, but studies have shown conflicting results [10] and the facts that girls perform better than boys in episodic memory [37,38] and that the difference seems to be consistent over the entire life span [9,39] argue against this explanation as the sole cause for gender differences.

The finding of an association between a higher BMI and impairment in both episodic and semantic memory in men is in line with previous studies that have found that obesity in middle life is associated with a future risk of poorer memory in older age [15,40,41].

A high circulating glucose concentration was, somewhat surprising, associated with better semantic memory in men. We have not found any other publication corroborating this finding; thus it must be tested in other studies to evaluate the robustness of this observation.

A major strength of the present study is the extensive memory tests performed in population-based memory study that were combined with the results from a population-based health survey. The latter included standardized oral glucose tolerance tests and questionnaires about cardiovascular events, hypertension, and lifestyle. Another strength in this study is the use of PLS as statistical method. PLS is able to deal with multicolinearity unlike for example multiple linear regression who assumes independence among measures. To eliminate the effect of multicolinearity makes it possible to remain a higher statistical power by isolating the effect of each potential influencing factor.

This study has some limitations. There was a time gap (<6 months) between collection of data in the studies that theoretically could allow changes in metabolic balance, but there was no apparent effect on episodic or semantic memory in our results due to this difference in time. The CES-D screening test for depression was missing in 48 (16%) participants, and it is possible that some of those participants were suffering from depression that might have affected memory functions. However, the exclusion of participants without information on CES-D did not alter the results.

The participants were asked about previous CVD and it was adjusted for in the statistical analysis, but no neuroimaging or neurological examination was made. Considering that 24% of the population reported previous CVD, it is possible that some of the participants had vascular changes that could affect the results. There was a potential for selection bias in the study population by linkage of two large population studies. However, when we compared education and weight between the 1346 persons in the first sample and the 291 persons in the study sample there was no significant difference. This is a cross-sectional study and, therefore, we are not able to study any direction of the effect of glucose on memory function. There was a fairly wide distribution of fasting plasma insulin in women. This may have led to false negative results. However, since the effect size of plasma insulin was so small it is not reasonable to assume that it has any relevant effect. Finally, HOMA-IR was used as a proxy for insulin resistance. A study using the gold standard, i.e., the hyperinsulinemic euglycemic clamp technique to further evaluate the effect of insulin and glucose on cognitive function would be of major interest. However, clamp techniques are not feasible in large epidemiological studies.

Over the past several decades, there has been a worldwide increase in diseases related to metabolic perturbations such as obesity, diabetes, and hypertension [42]. With a growing number of older persons, the impact of these conditions will have an increasingly significant role in health care. Metabolic changes are unlike many other risk factors for dementia and memory impairment because they are preventable with lifestyle changes or drug treatments. Thus, our study focuses on the association between early stages of metabolic perturbations and memory dysfunction because the early stages represent a window of opportunity for preventive measures.

## Conclusions

In conclusion, we found no significant association between fasting insulin concentrations or insulin resistance and episodic or semantic memory. We found a negative association between fasting glucose concentration and episodic memory in women. This suggests that elevated glucose concentrations could be important as a modifier of episodic memory in a middle-aged non-diabetic population. This is likely to be through interactions with other metabolic and age-related processes that are already in progress.
